# The analytical and clinical validity of AI algorithms to score TILs in TNBC: can we use different machine learning models interchangeably?

**DOI:** 10.1016/j.eclinm.2024.102928

**Published:** 2024-11-15

**Authors:** Joan Martínez Vidal, Nikos Tsiknakis, Johan Staaf, Ana Bosch, Anna Ehinger, Emma Nimeus, Roberto Salgado, Yalai Bai, David L. Rimm, Johan Hartman, Balazs Acs

**Affiliations:** a Department of Oncology and Pathology, Karolinska Institutet, Stockholm, Sweden; b Division of Oncology, Department of Clinical Sciences Lund, Lund University, Medicon Village, SE-22381, Lund, Sweden; c Department of Hematology, Oncology and Radiation Physics, Region Skåne, Lund, Sweden; d Department of Genetics, Pathology and Molecular Diagnostics, Laboratory Medicine, Region Skåne, Lund, Sweden; e Division of Surgery, Department of Clinical Sciences, Lund University, Lund, Sweden; f Department of Surgery, Skåne University Hospital, Malmö, Sweden; g Department of Pathology, GZA-ZNA Hospitals, Antwerp, Belgium; h Division of Research, Peter MacCallum Cancer Centre, Melbourne, Australia; i Department of Pathology, Yale School of Medicine, New Haven, CT, USA; j Department of Internal Medicine (Medical Oncology), Yale University School of Medicine, New Haven, CT, USA; k Department of Clinical Pathology and Cancer Diagnostics, Karolinska University Hospital, Stockholm, Sweden

**Keywords:** TILs, Tumor infiltrating lymphocytes, Breast cancer, Machine learning, Deep learning, Artificial intelligence

## Abstract

**Background:**

Pathologist-read tumor-infiltrating lymphocytes (TILs) have showcased their predictive and prognostic potential for early and metastatic triple-negative breast cancer (TNBC) but it is still subject to variability. Artificial intelligence (AI) is a promising approach toward eliminating variability and objectively automating TILs assessment. However, demonstrating robust analytical and prognostic validity is the key challenge currently preventing their integration into clinical workflows.

**Methods:**

We evaluated the impact of ten AI models on TILs scoring, emphasizing their distinctions in TILs analytical and prognostic validity. Several AI-based TILs scoring models (seven developed and three previously validated AI models) were tested in a retrospective analytical cohort and in an independent prospective cohort to compare prognostic validation against invasive disease-free survival endpoint with 4 years median follow-up. The development and analytical validity set consisted of diagnostic tissue slides of 79 women with surgically resected primary invasive TNBC tumors diagnosed between 2012 and 2016 from the Yale School of Medicine. An independent set comprising of 215 TNBC patients from Sweden diagnosed between 2010 and 2015, was used for testing prognostic validity.

**Findings:**

A significant difference in analytical validity (Spearman's r = 0.63–0.73, p < 0.001) is highlighted across AI methodologies and training strategies. Interestingly, the prognostic performance of digital TILs is demonstrated for eight out of ten AI models, even less extensively trained ones, with similar and overlapping hazard ratios (HR) in the external validation cohort (Cox regression analysis based on IDFS-endpoint, HR = 0.40–0.47; p < 0.004).

**Interpretation:**

The demonstrated prognostic validity for most of the AI TIL models can be attributed to the intrinsic robustness of host anti-tumor immunity (measured by TILs) as a biomarker. However, the discrepancies between AI models should not be overlooked; rather, we believe that there is a critical need for an accessible, large, multi-centric dataset that will serve as a benchmark ensuring the comparability and reliability of different AI tools in clinical implementation.

**Funding:**

Nikos Tsiknakis is supported by the 10.13039/501100004359Swedish Research Council (Grant Number 2021-03061, Theodoros Foukakis). Balazs Acs is supported by The 10.13039/501100003748Swedish Society for Medical Research (Svenska Sällskapet för Medicinsk Forskning) postdoctoral grant. Roberto Salgado is supported by a grant from 10.13039/100001006Breast Cancer Research Foundation (BCRF).

**Research in context**

**Evidence before this study**

In the last years, tumor-infiltrating lymphocytes (TILs) have become increasingly significant, showcasing their predictive and prognostic potential for early and metastatic triple-negative breast cancer (TNBC). However, TILs remain a semi-quantitative biomarker that is susceptible to inter-observer variability. Over the last couple of years, different machine-learning approaches have been proposed to score anti-tumor immunity to overcome low-scoring variability (PubMed search: TILs + artificial intelligence, 2024026). There are many histopathology-focused AI systems commercially available, yet the adoption of computational pathology has been minimal. Although software manufacturers provide validation to regulatory bodies to prove the use of the AI tool necessary for their approval (FDA clearance or CE marking as an example), independent validation studies mimicking the challenges of real-life clinical practice are lacking. Furthermore, there are no studies comparing various AI models focusing on both analytical and prognostic validity.

**Added value of this study**

We show evidence of variability among ten AI-based TILs scoring models with respect to their analytical validity when considering both internal and external validation cohorts. Regarding prognostic validity, eight out of ten models were found to be statistically significant in an independent prospective cohort against invasive disease-free survival, with similar and overlapping hazard ratios. Interestingly, even models with fewer training samples exhibited robust prognostic potential compared to similar methods trained on larger datasets.

**Implications of all the available evidence**

We believe that the prognostic robustness of the host anti-tumor immunity as a biomarker (measured by TILs), may lead to even less extensively trained models, performing comparably well in assessing outcomes. However, this may result in models that have overfitted to their respective training datasets, limiting their applicability in clinical practice. Our study demonstrates the need for comparability between different AI methodologies before clinical implementation.

## Introduction

The last five years have borne witness to the unprecedented development of novel therapies for early-stage breast cancer.^1^^,^^2^ There is an urgent need for systematic implementation of biomarker-driven risk stratification to avoid over- and under-treatment to accurately select patients who will benefit from additional therapy. The quantity of lymphocytes infiltrating the tumoral stroma (stromal tumor-infiltrating lymphocytes, sTILs) on standard haematoxylin and eosin (H&E) stained formalin-fixed paraffin embedded (FFPE) tissue is known to be a robust prognostic feature in early-stage triple-negative breast cancer (TNBC)^3^, ^4^, ^5^, ^6^, ^7^, ^8^ where high levels of sTILs effectively down-stage clinical risk compared to staging according to clinicopathological features alone.^9^ The International Immuno-Oncology Biomarker Working Group on Breast Cancer (TIL-WG) has produced guidelines to standardize sTIL assessment with demonstrable reproducibility.^5^^,^^10^, ^11^, ^12^ However, inter-observer variability remains inevitable as sTILs scoring is a semi-quantitative biomarker that is inherently limited in its capacity to capture the complexity of tumor-immune microenvironment (TME).

Computational analysis of digital pathology images through machine learning algorithms can address standardization issues and has the potential to learn from image-based features that exist beyond the perception of the human eye. This additional precision and spatial detail may yield more complex and robust predictions than simple quantification of TILs.^13^, ^14^, ^15^, ^16^

Demonstrating robust analytical and clinical validity are the key challenges currently preventing the effective integration of AI-TILs models into clinical workflows. Unaddressed, this poses an unacceptable risk when it comes to clinical decision-making.^17^, ^18^, ^19^ Access to high-quality, high-volume training, test and validation datasets is critical to avoid “over-fitted” models that only perform well on data with certain features and lack external validity.^20^^,^^21^ Furthermore, there is still insufficient evidence regarding how different AI models affect TILs assessment distinctly. To address these issues, this study aims to compare the analytical and prognostic performance of ten AI-based TIL assessment models. The gold standard of this study regards manual sTILs scoring from two expert pathologists.

## Methods

### Patient cohorts

#### Yale cohort: analytical validity

Diagnostic whole tissues section slides (WTS) of 106 women with surgically resected primary invasive (stage I-III) TNBC tumors diagnosed between 2012 and 2016 were obtained from the Yale School of Medicine Department of Pathology archives. The data were retrieved under permission from the Yale Human Investigation Committee protocol #9505008219 to DLR.^22^ A selection of one digital slide was considered for four patients with duplicate slides based on the presence of artifacts and invasive tumor areas. Thirteen slides were excluded due to the absence of invasive tumor or the presence of extensive artifacts. Finally, 92 digital slides of 79 patients (Table 1) were used for training (N = 50) and internal testing of the analytical performance of the models (N = 42). All slides were stratified at the patient level to prevent any data leakage between the training and internal validation sets.

**Table 1:** Clinical characteristics of patients included in the study.

	Yale cohort	Swedish cohort	p-value^b^
**Cases**	79^a^	215	–
**Age**			
<50	21 (26.6%)	49 (23%)	0.6
≥50	58 (73.4%)	166 (77%)	
**Tumor size**			
≤20	37 (46.8%)	108 (50.2%)	0.7
>20	42 (53.2%)	107 (49.8%)	
Continuous (mm)	21 (15–30.5)	21 (15–30)	0.78
**Histologic grade**			
2	17 (21.5%)	22 (10.2%)	0.02
3	61 (77.2%)	190 (88.4%)	
NA	1 (1.3%)	3 (1.4%)	
**Nodal status**			
Negative	52 (65.8%)	132 (61.4%)	0.64
Positive	27 (34.2%)	81 (37.7%)	
NA	–	2 (0.9%)	
**Chemotherapy**			
No	12 (15.2%)	57 (26.5%)	0.46
Yes	46 (58.2%)	158 (73.5%)	
NA	21 (26.6%)	–	
**Follow-up (years)**			
Follow-up time	10.63 (6.3–16.4)	3.94 (3.2–5.32)	–

For dichotomous variables, number of samples and prevalence percentages are reported.

For continuous variables, median as well as first and third quartiles are reported.

a EQC cohort: 92 slides from 79 patient cases.

b Chi-squared test for categorical variables and Mann–Whitney test for continuous variables.

#### Swedish cohort: clinical validity

A Swedish cohort comprising WTS and clinical data from 215 women with surgically resected TNBC was used as an independent external cohort to validate the prognostic performance of all models. The patients included were enrolled in the prospective, observational, population-based SCAN-B study (ClinicalTrials.gov ID NCT02306096)^23^^,^^24^ between 2010 and 2015 and has been reported previously^25^ (Table 1). The SCAN-B study was approved by the Regional Ethical Review Board in Lund, Sweden (applicable registration numbers 2009/658, 2015/277, 2016/742, 2018/267, and 2019/01252). Three patients were excluded from the analysis due to image processing problems. Since TNBC cases are just 10–15% of breast cancer patients,^26^ we believe that the cohorts of our study, especially the external validation one with 215 patients, are representative of the TNBC population. Furthermore, the only variable showing a statistically significant difference between cohorts was grade, which is also subject to high variability between pathologists^27^ (Table 1).

### Digital-image analysis

#### Image data acquisition and processing techniques

Whole slide scanning of stained slides was performed at 20× using the Leica Aperio ScanScope, Controller v10.2.0.2359 and ScanScope Console v10.2.0.2352 imaging software.^22^ For the WTS Swedish cohort, H&E-stained slides were digitized using the NanoZoomer 2.0-HT (Hamamatsu Photonics K.K.) platform at 20×, with a pixel size of 0.4537 × 0.4537 mm.^25^

#### Supervised machine learning models

**Fig. 1:**
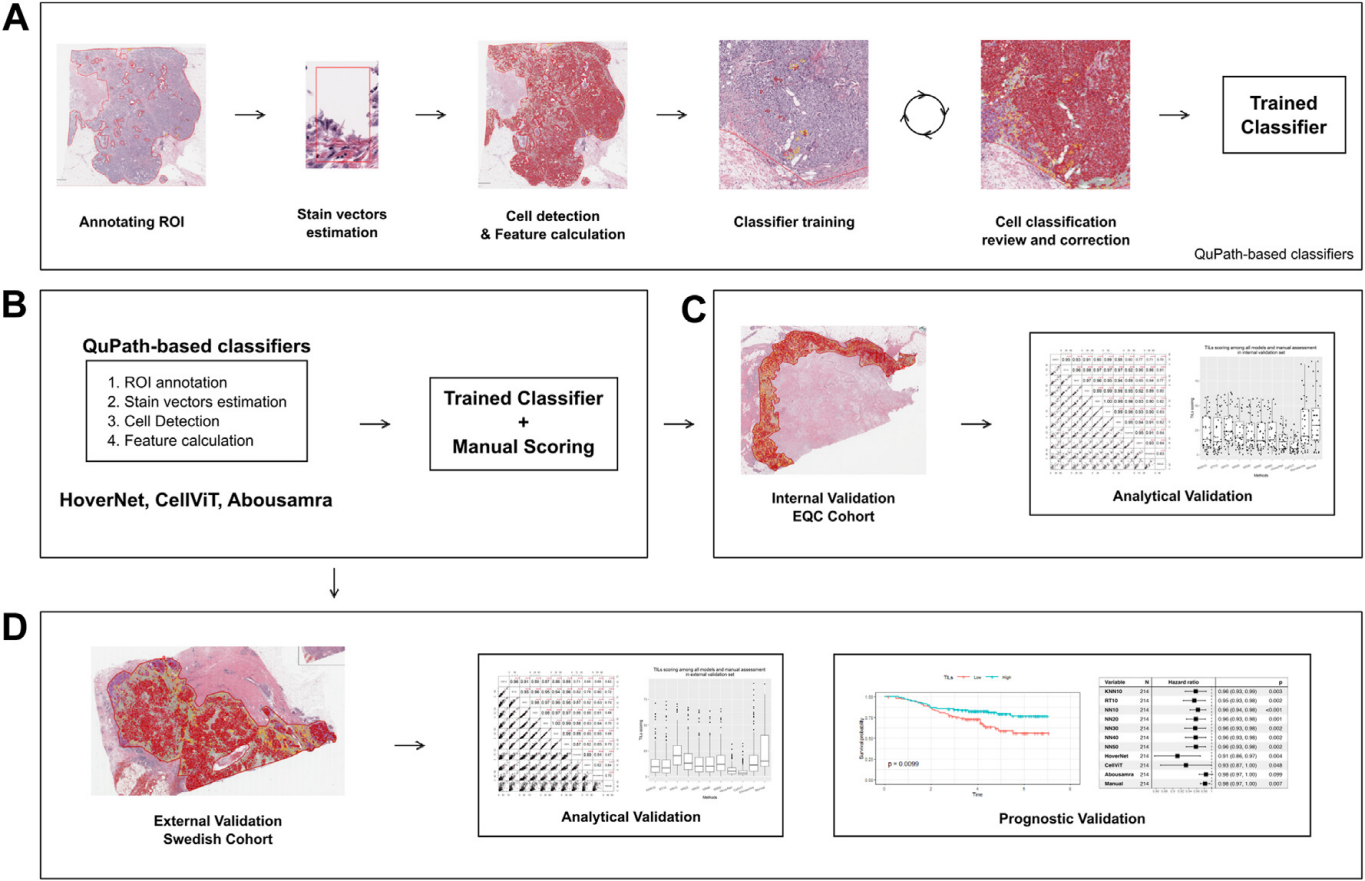
Digital image analysis flowchart for classifiers development and utilization. (a) Preprocessing and classifiers training pipeline (KNN10, RT10, NN10, NN20, NN30, NN40 and NN50). (b) Application of TILs models. (c) Analytical evaluation of the classifiers on the Yale internal validation set. (d) Prognostic evaluation in an independent validation set. Note that the “trained classifier” applied in sub-figures b-d is the one created in a, in addition to HoverNet, CellViT and Abousamra's.

The QuPath open-source software platform (version 0.3.2) was used to build automated TILs scoring algorithms.^28^ A flowchart for the quantitative analysis of tissue images is illustrated in Fig. 1. First, regions of interest (ROI) including invasive cancer areas were drawn in QuPath following the guideline proposed by the ITWG^11^ as follows: (i) Include TILs within the borders of the invasive tumor, including both “central tumor” and “invasive margin”. (ii) All mononuclear cells (including lymphocytes and plasma cells) should be scored, but polymorphonuclear leukocytes are excluded. (iii) Exclude TILs at a distance outside of the tumor borders. (iv) Exclude TILs around in situ regions and normal lobules. (v) Exclude areas with crush artifacts, necrosis, and regressive hyalinization. *Stain-vectors estimation* was performed for each slide to obtain a normalized representation of each stain-component. *Watershed cell detection* was used to segment the cells in the image with the default settings. Smoothed object features at 25 and 50 μm radius were computed to supplement the existing measurements of individual cells. Models from three different families of models (K Nearest Neighbor–KNN, Random Trees -RT, Neural Network–NN) were trained on a subset of 10 training images. Based on the evaluation of those models, additional training scenarios were explored, training the best performing model on an increasing number of patient samples (i.e. using 20, 30, 40 and 50 samples). For clearer reference, the naming of each method complies with the following pattern: MN, where M is the name of the method and N is the number of training samples used. The clinical characteristics of all patients included in each training set of the NN10-50 classifiers, as well as the internal validation subset of the Yale cohort are presented in Supplementary Figure S1.

The training of each classifier was done based on a human-in-the-loop strategy, while multiple rounds of review and correction were required to achieve optimal performance. At first, each image contained manual annotations for approximately 450 cells, with a minimum number of 150 tumor cells and 150 immune cells (lymphocytes). The rest 150 cells were both classified as stroma or “other” subtypes (including necrosis and epithelial cells, among others). All the cells were selected individually upon the entire invasive tumor area, ensuring heterogeneity throughout the sample was considered. Therefore, different annotations were used to ensure unbiased selection of region of interests (ROIs) with respect to each classifier. This decision was based on the assumption that each classifier would have been partly biased by the interactive training of the immediate previously trained one, if there would have been overlapping annotated ROIs. Additional annotations were added at each step if adequate classification performance was not met. Adequate classification performance was set to be an accuracy of 95% within QuPath's classifier training module. This step was performed by JMV, being supervised by expert pathologists BA and JH.

#### Deep-learning modes

Three deep learning models were included in the study as independent predictors, i.e. HoverNet,^29^ CellViT^30^ and Abousamra et al.^31^ The former two regard cell segmentation Convolutional Neural Network (CNN) models that detect and phenotype cells. Both have been pretrained on the PanNuke dataset,^32^ encompassing nearly 200,000 nuclei across 19 tissue types, including breast. The predicted classes include neoplastic epithelial, inflammatory, connective, necrotic, and non-neoplastic epithelial cells, which were transformed into tumor, immune, stroma, and other cells for TILs scoring in QuPath. On the other hand, Abousamra's model was trained on 23 cancer types for tile-level classification (i.e. whether small image patches–50 × 50 μm^2^–contain lymphocyte cells or not) rather than cell detection.^31^ While there is a significant methodological disparity between Abousamra's model and the rest, we believe that including it as a baseline independent predictor is valuable for the study.

#### TILs scoring

The easTILs formula was used to calculate digital TILs scoring^14^ for the models that are based on cell detection (i.e. all but Abousamra's). The mathematical formula is described as easTILs = 100 ∗ [sum of TILs area (mm^2^)/stroma area (mm^2^)], where stroma area (mm^2^) = sum of tumor region areas analyzed (mm^2^)—sum of tumor cell area (mm^2^). For Abousamra's model, the percentage of invasive cancer region predicted as lymphocyte patches is calculated and used as TILs scoring. The pathologists' assessment of sTILs scores was conducted on a non-overlapping subset of the data, with BA evaluating one half and JH evaluating the other, following international guidelines for TILs assessment.

#### Statistical analysis

**Fig. 2:**
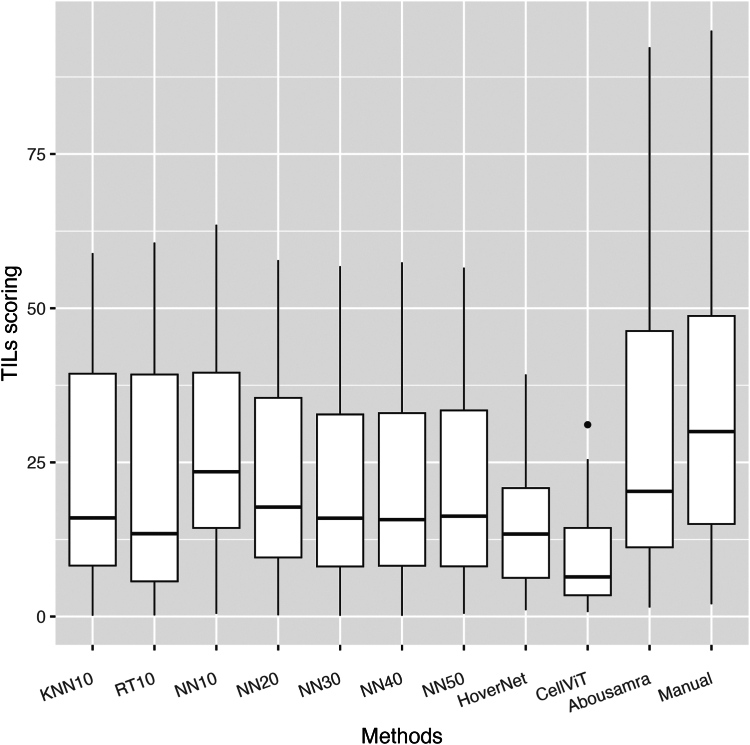
Boxplots of all TILs scoring methods in the internal validation Yale cohort. The horizontal black line in the boxplots indicates the median, the outlined solid box represents the 25th–75th percentile, the black vertical lines represent the range of the data distribution and dots are outliers from the distribution.

**Fig. 3:**
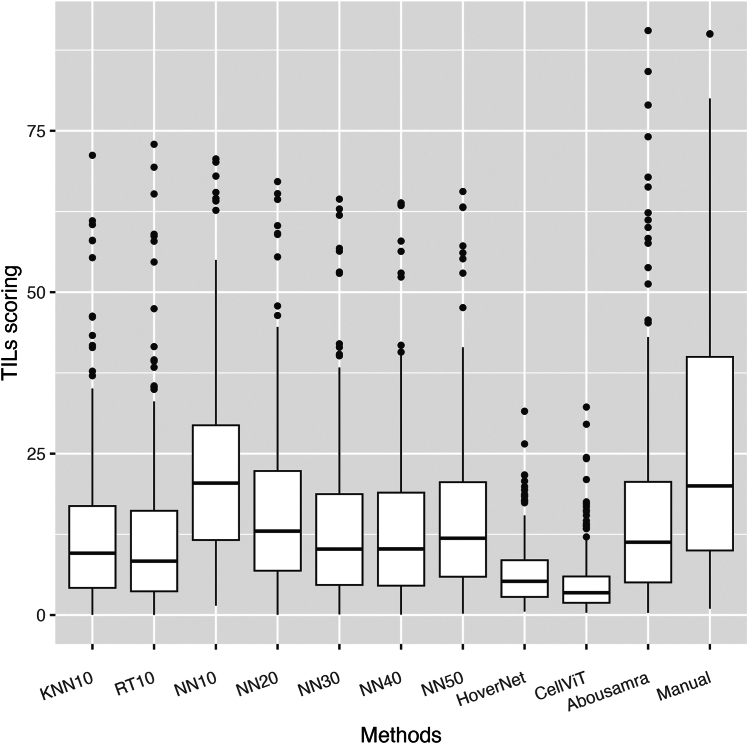
Boxplots of all TILs scoring methods in the external SCAN-B validation cohort. The horizontal black line in the boxplots indicates the median, the outlined solid box represents the 25th–75th percentile, the black vertical lines represent the range of the data distribution and dots are outliers from the distribution.

**Supplementary Figure S2:**
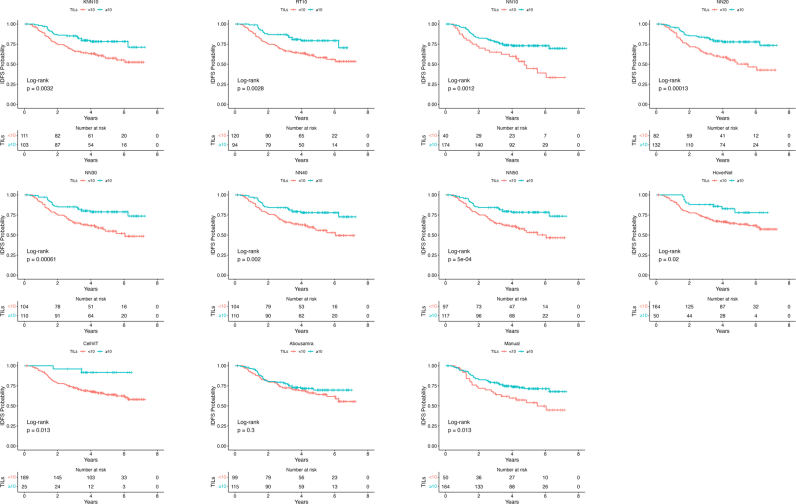
Kaplan Meier curves for all scoring methods dichotomized at 10% value, using IDFS as clinical endpoint, in the SCAN-B validation cohort. The red curve regards the Low-TILs subgroup (<10%), while the blue curve regards the High-TILs subgroup (≥10%).

**Supplementary Figure S3:**
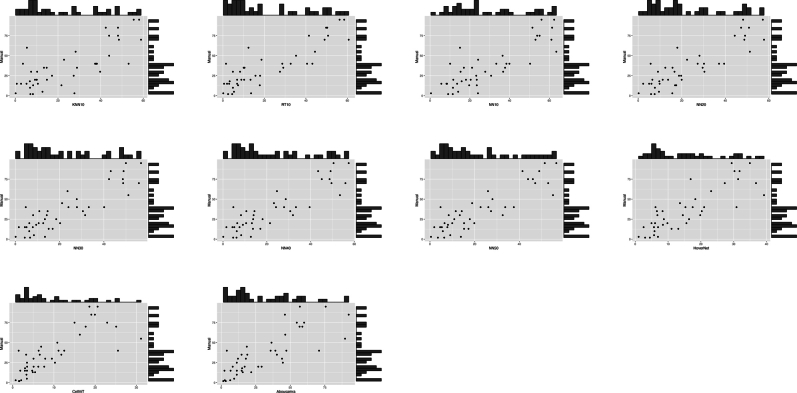
Scatter and histogram plots of each AI-based TILs score against manual assessment of sTILs in the internal validation set of Yale cohort. The central plot of each subfigure shows the bivariate scatter plot, while the top and right histograms illustrate the distributions of each method compared to the manual sTILs.

**Supplementary Figure S4:**
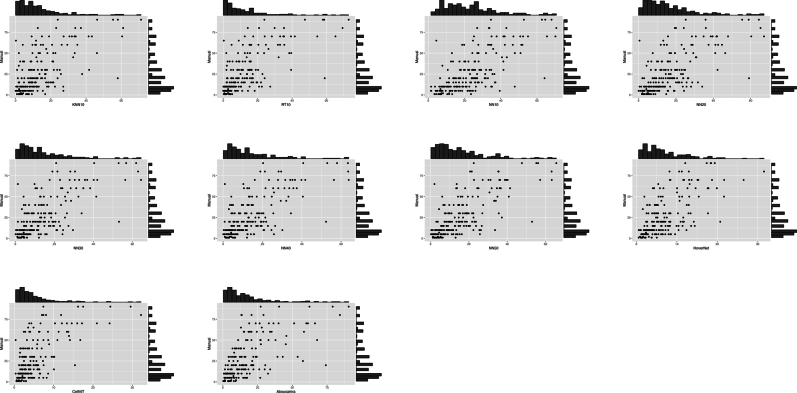
Scatter and histogram plots of each AI-based TILs score against manual assessment of sTILs in the external SCAN-B validation cohort. The central plot of each subfigure shows the bivariate scatter plot, while the top and right histograms illustrate the distributions of each method compared to the manual sTILs.

**Supplementary Figure S5:**
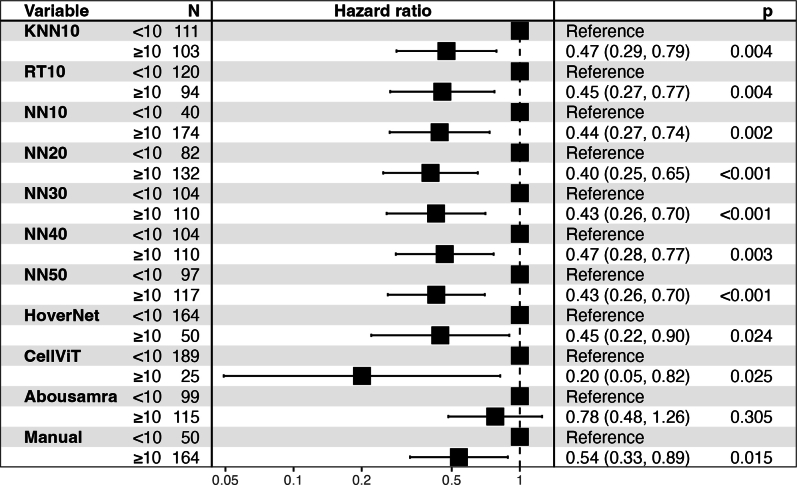
Forest plot for the univariate Cox analysis of all TIL scoring methods dichotomized at 10% value, using IDFS as clinical endpoint, in the SCAN-B validation cohort. The black squares regard the hazard ratio values, while the horizontal error bars indicate the confidence interval (CI). The CI is also shown in parentheses next to the hazard ratio value. The number range at the bottom of the plot regards the hazard ratio values, while the dotted vertical line pinpoints the point where HR=1.

**Supplementary Figure S6:**
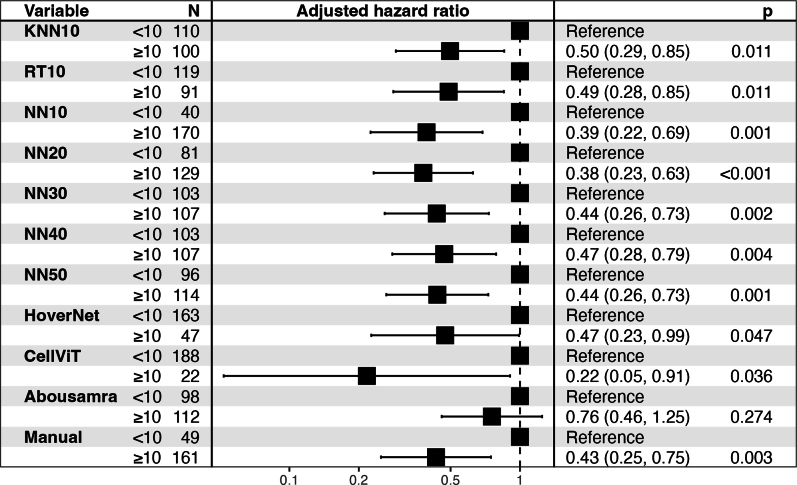
Forest plot for the multivariate Cox analysis of all TIL scoring methods dichotomized at 10% value (adjusted for age group, tumor size group, grade and nodal status), using IDFS as clinical endpoint, in the SCAN-B validation cohort. The black squares regard the hazard ratio values, while the horizontal error bars indicate the confidence interval (CI). The CI is also shown in parentheses next to the hazard ratio value. The number range at the bottom of the plot regards the hazard ratio values, while the dotted vertical line pinpoints the point where HR=1.

**Supplementary Figure S7:**
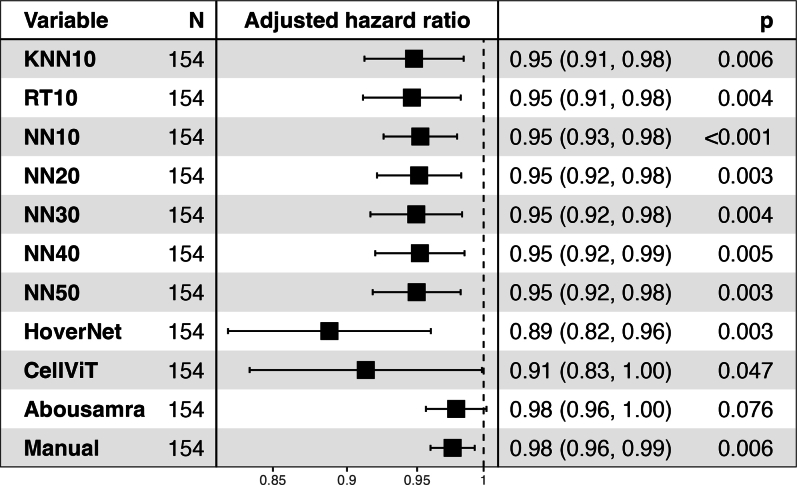
Forest plot for the multivariate Cox analysis of all continuous TIL scoring methods (adjusted for age group, tumor size group, grade and nodal status), using IDFS as clinical endpoint, in the chemotherapy-administered subgroup of the SCAN-B validation cohort. The black squares regard the hazard ratio values, while the horizontal error bars indicate the confidence interval (CI). The CI is also shown in parentheses next to the hazard ratio value. The number range at the bottom of the plot regards the hazard ratio values, while the dotted vertical line pinpoints the point where HR=1.

**Supplementary Figure S8:**
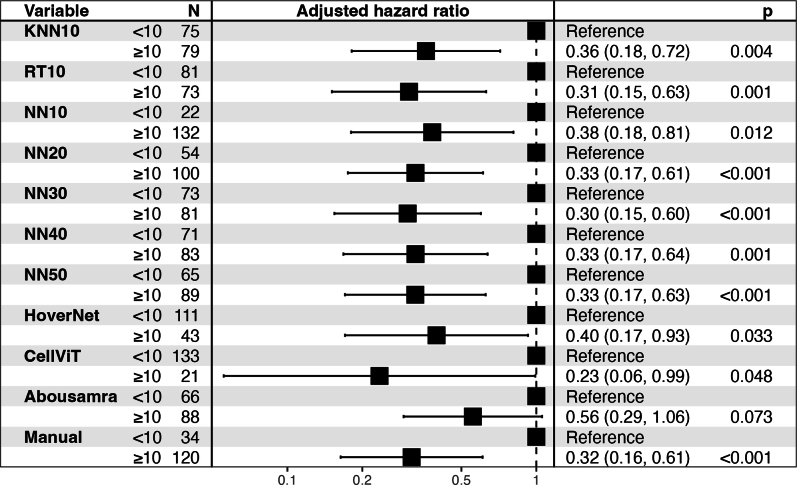
Forest plot for the multivariate Cox analysis of all TIL scoring methods dichotomized at 10% value (adjusted for age group, tumor size group, grade and nodal status), using IDFS as clinical endpoint, in the chemotherapy-administered subgroup of the SCAN-B validation cohort. The black squares regard the hazard ratio values, while the horizontal error bars indicate the confidence interval (CI). The CI is also shown in parentheses next to the hazard ratio value. The number range at the bottom of the plot regards the hazard ratio values, while the dotted vertical line pinpoints the point where HR=1.

**Supplementary Figure S9:**
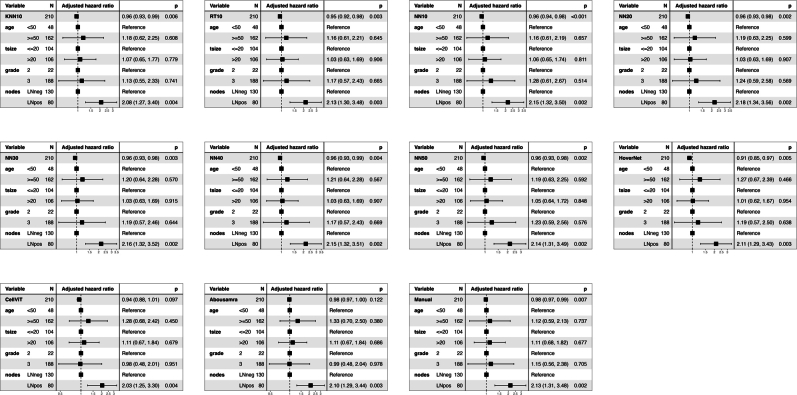
Forest plot for the multivariate Cox analysis of continuous TILs scores of all methods (adjusted for age group, tumor size group, grade and nodal status), using IDFS as clinical endpoint, in the SCAN-B validation cohort. Adjusted covariates which were omitted in the main manuscript have been included here for reference. Similar HR values for each covariate is exhibited in dichotomized and chemo-administered analyses. The black squares regard the hazard ratio values, while the horizontal error bars indicate the confidence interval (CI). The CI is also shown in parentheses next to the hazard ratio value. The number range at the bottom of the plot regards the hazard ratio values, while the dotted vertical line pinpoints the point where HR=1.

For evaluating the analytical performance of the models, classification metrics were used based on 2500 annotated cells across 5 slides. Spearman's correlation coefficient was computed to evaluate the correlation of the manual sTIL score of the pathologists and the easTIL scores of each AI-based model based on both the internal and external validation sets. Invasive disease-free survival (IDFS) was defined according to STEEP guidelines,^33^ as the time from diagnosis to either death of any cause or invasive breast-cancer related events (loco-regional and distant recurrence). TILs scoring measurements were dichotomized for Kaplan–Meier plots based on a 10% cut-off^34^, ^35^, ^36^, ^37^, ^38^ and presented as supplementary files (Supplementary Figure S2), with differences in survival times being tested using the log-rank test. However, TILs scoring measurements are utilized as continuous variables for Cox regression analysis in the main manuscript, due to the inherent differences in their distributions (Figs. 2 and 3). Scatter and histogram plots of TILs scores of each method against manual assessment of sTILs are also presented for the internal and external validation sets in Supplementary Figures S3 and S4 respectively. Univariate and multivariate Cox regression analyses were performed to assess the prognostic value of TILs scores when adjusted for key clinicopathological variables, i.e. age, tumor size, histologic grade, and nodal status. Age and tumor size variables are used in a dichotomized form based on cut-off values of 50 years and 20 mm respectively. The results of Cox proportional hazard models are shown in forest plots as hazard ratio (HR) with 95% confidence interval (CI) and Wald test p-values. Forest plots for dichotomized TILs scores are presented in supplementary files (Supplementary Figures S5 and S6). Forest plots for the chemotherapy-administered subgroup (N = 158–73.5% of the external validation cohort) are also presented in supplementary files (Supplementary Figures S7 and S8). For the sake of conserving space, hazard ratios for adjusted variables were omitted from the multivariate forest plots, as the primary focus of this study is the effect of TILs scoring and all covariates are established biomarkers in TNBC but are presented in Supplementary Figure S9 for reference. The proportional hazard assumption has been checked with the cox.zph function of R's survival package (version 3.5.8) and is maintained for all machine-read TILs variables. The level of significance for all statistical tests is set at 5%. Performance evaluation and statistical analyses were performed using Python version 3.9 and R version 4.3.2 software.

### Role of funding

The funder of the study had no role in the design and conduct of the study; collection, management, analysis, and interpretation of the data; preparation, review, or approval of the manuscript; and decision to submit the manuscript for publication.

## Results

### Analytical validity

**Fig. 4:**
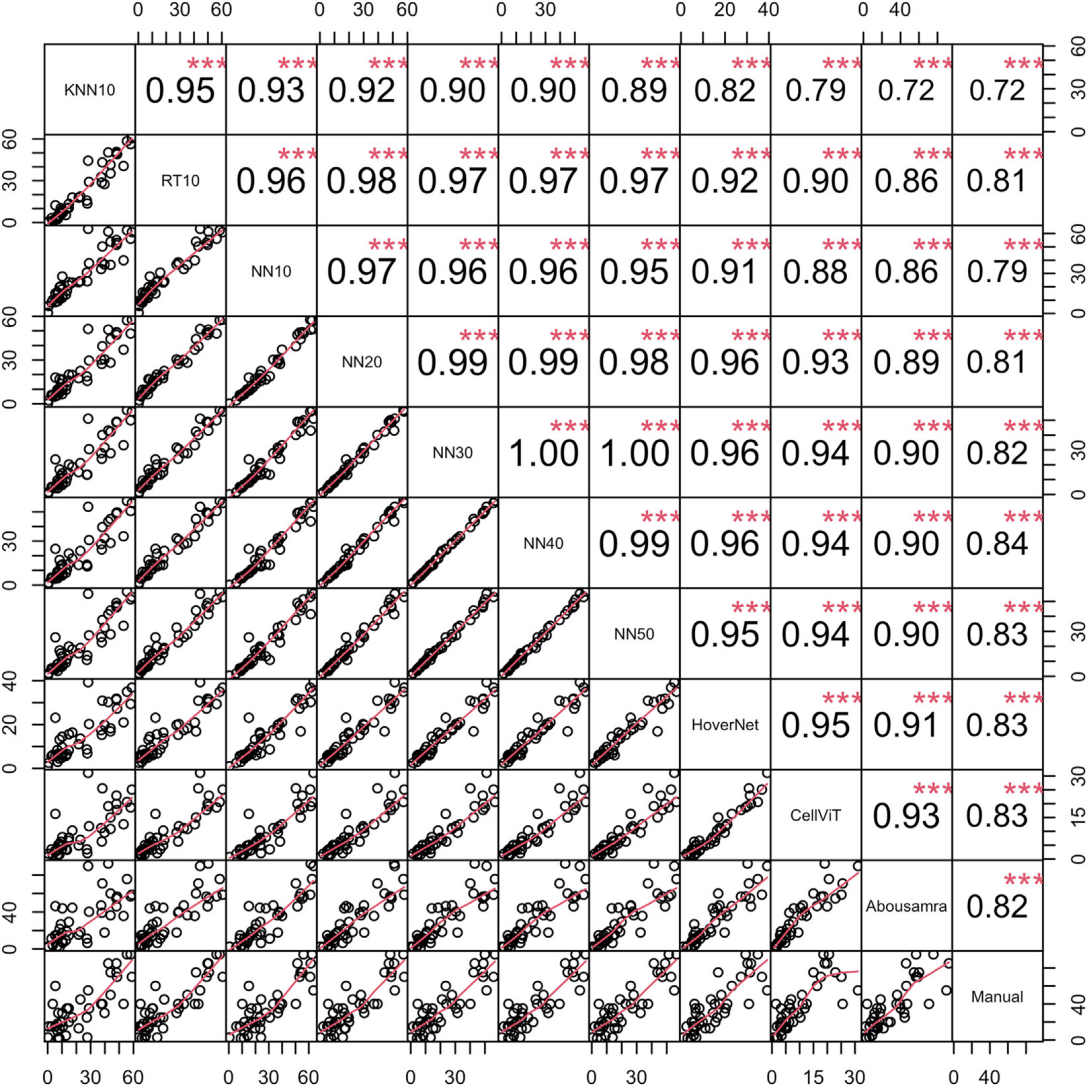
Spearman's correlation coefficient matrix for all methods and manual sTILs in the internal validation set of Yale cohort. The bottom part of the diagonal shows the bivariate scatter plots with a fitted line. The upper part of the diagonal shows the correlation coefficient value and the significance level as stars. The three stars correspond to a p-value <0.001.

Considering the evaluation of the analytical performance of the models, we first report the findings on the internal validation set of the Yale cohort. KNN10 and RT10 showed the widest TILs score distribution, while NN-based models showed similar and consistent distributions of TILs scoring amongst themselves (Fig. 2). On the other hand, HoverNet- and CellViT-based measurements exhibited the narrowest distributions, while Abousamra's exhibited the widest distribution, like Manual sTILs (Fig. 2). The correlation of TILs scoring against manual sTILs assessment varied across models (Fig. 4). KNN10 correlated moderately with sTILs (*r* = *0.72,* p < *0.001*), while NN10 achieved slightly better results (*r* = *0.79,* p < *0.001*). RT10 achieved the best correlation amongst the models trained on a limited number of patient samples (*r* = *0.81,* p < *0.001*). Increasing the number of training samples in the model training resulted in gradually increasing correlation coefficient with the gold standard pathologist-read sTILs (*NN20: r* = *0.81,* p < *0.001; NN30: r* = *0.82,* p < *0.001; NN40: r* = *0.84,* p < *0.001; NN50: r* = *0.83,* p < *0.001*). HoverNet and CellViT achieved the second highest correlation with sTILs (*r* = *0.83,* p < *0.001*). Finally, Abousamra's model resulted in good correlation as well (*r* = *0.82,* p < *0.001*).

**Fig. 5:**
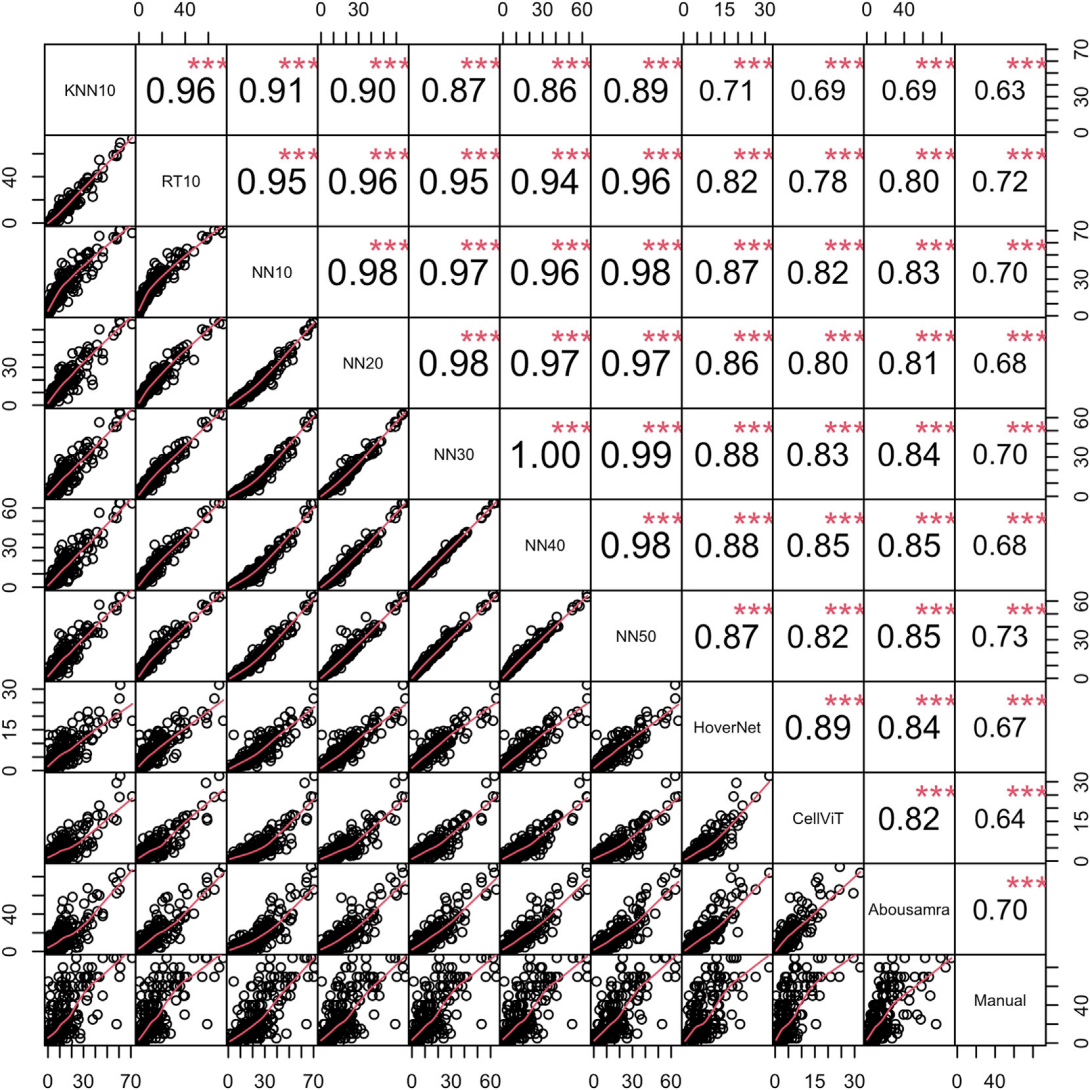
Spearman's correlation coefficient matrix for all methods and manual sTILs in the external SCAN-B validation cohort. The bottom part of the diagonal shows the bivariate scatter plots with a fitted line. The upper part of the diagonal shows the correlation coefficient value and the significance level as stars. The three stars correspond to a p-value <0.001.

To have an unbiased overview of all TILs scoring methods in terms of their analytical performance, we also present the boxplot figure and correlation matrix as calculated in the external SCAN-B validation cohort (Figs. 3 and 5 respectively). The distributions of TILs scores of all methods were much narrower and the median scores were smaller in the external validation cohort than in the internal cohort (Fig. 3). All correlation coefficients decreased in value, with KNN10 still showing the lowest correlation with manual sTILs (*r* = *0.63,* p < *0.001*). NN50 and RT10 performed the best (NN50: *r* = *0.73,* p < *0.001; RT10: r* = *0.72,* p < *0.001*). However, increasing the number of training samples did not improve the correlation with pathologist-read sTILs (*NN10: r* = *0.70,* p < *0.001; NN20: r* = *0.68,* p < *0.001; NN30: r* = *0.70,* p < *0.001; NN40: r* = *0.68,* p < *0.001; NN50: r* = *0.73,* p < *0.001*). The deep learning-based CellViT, HoverNet, and Abousamra's models showed comparable correlation indices with pathologist-read sTILs (*CellViT: r* = *0.64,* p < *0.001; HoverNet: r* = *0.67,* p < *0.001;* Abousamra's: *r* = *0.70,* p < *0.001*), similarly to the KNN, NN and RT models.

### Clinical validity

**Fig. 6:**
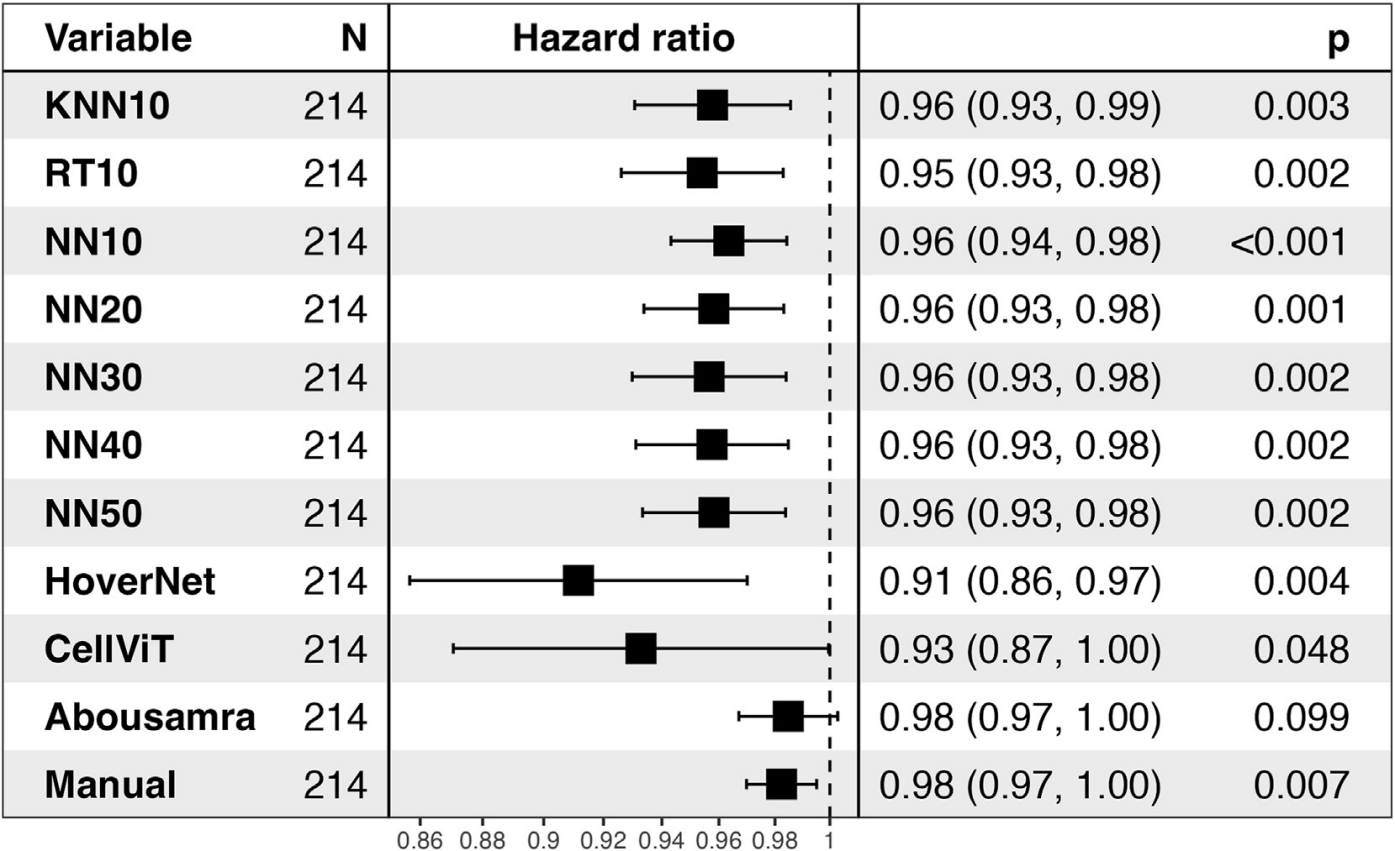
Forest plot for the univariate Cox analysis of continuous TILs scores of all methods, using IDFS as clinical endpoint, in the SCAN-B validation cohort. The black squares regard the hazard ratio values, while the horizontal error bars indicate the confidence interval (CI). The CI is also shown in parentheses next to the hazard ratio value. The number range at the bottom of the plot regards the hazard ratio values, while the dotted vertical line pinpoints the point where HR = 1.

**Fig. 7:**
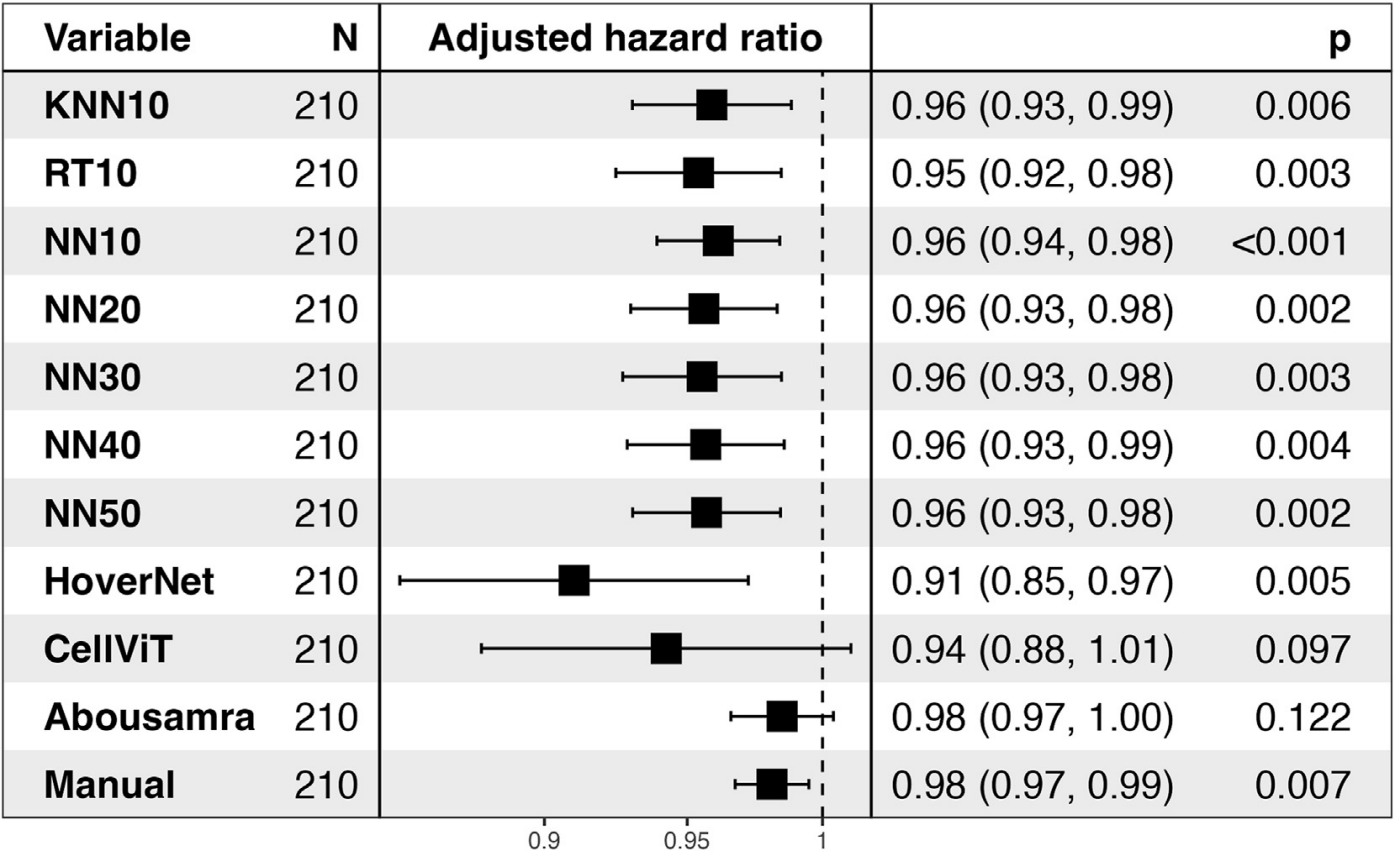
Forest plot for the multivariate Cox analysis of continuous TILs scores of all methods (adjusted for age group, tumor size group, grade and nodal status), using IDFS as clinical endpoint, in the SCAN-B validation cohort. Hazard ratios for the adjusted variables are not illustrated to conserve space. The black squares regard the hazard ratio values, while the horizontal error bars indicate the confidence interval (CI). The CI is also shown in parentheses next to the hazard ratio value. The number range at the bottom of the plot regards the hazard ratio values, while the dotted vertical line pinpoints the point where HR = 1.

Associations of models and patient outcome were investigated in the external Swedish SCAN-B validation cohort using IDFS as clinical endpoint. Kaplan–Meier plots with log-rank test showed significant differences in most, but not all, models between the low and high TIL patient groups subgroups (i.e. low vs high TILs based on a 10% cut-off) regarding IDFS endpoint (Supplementary Figure S2). Moreover, the models with significant results achieved a very robust HR (0.40–0.47) at 10% cut-off, except CellVit (HR = 0.20) (Supplementary Figure S5; Adjusted HRs for the dichotomized scores are illustrated in Supplementary Figure S6). When considering a univariate Cox regression analysis using continuous TILs scoring measurements due to the different scoring distributions found in the analytical setting, all models except Abousamra's (HR = 0.98, 95% CI = [0.97–1.0], p = 0.099) achieved statistically significant results (Fig. 6). All the significant models (9 out of 10) showed comparable and overlapping hazard ratios as follows: HoverNet HR = 0.91 (95% CI = [0.86–0.97], p = 0.004), CellViT HR = 0.93 (95% CI = [0.87–1.0], p = 0.048), KNN10 HR = 0.96 (95% CI = [0.93–0.99], p = 0.003), RT10 HR = 0.95 (95% CI = [0.93–0.98], p = 0.002), NN10 HR = 0.96 (95% CI = [0.94–0.98], p < 0.001). Increasing the training samples resulted in similar hazard ratios (Fig. 6), with NN50 exhibiting a HR = 0.96 (95% CI = [0.93–0.98], p = 0.002). For reference, manual sTILs assessment resulted in a HR = 0.98 (95% CI = [0.97–1.00], p = 0.007). When adjusting for age group, tumor size group, histologic grade, and nodal status, all models achieved similar results to their univariate counterparts (Fig. 7). However, CellViT and Abousamra models showed borderline non-significant results with a HR = 0.94 (95% CI = [0.88–1.01], p = 0.097) and HR = 0.98 (95% CI = [0.97–1.00], p = 0.122) respectively. All models showed similar adjusted HR values for the chemotherapy-administered subgroup of the validation cohort, with all but Abousamra model being significant, for both continuous and dichotomized TILs scores (Supplementary Figures S7 and S8).

## Discussion

There are many histopathology-focused AI systems commercially available, yet computational pathology has been slow to be widely adopted. While good-quality digitized tissue slides have been around for many years and received FDA approval in 2017, the adoption by pathology laboratories has been minimal. Although software manufacturers provide validation to regulatory bodies to prove the use of the AI tool necessary for their approval (FDA clearance or CE marking as an example), running large-scale studies mimicking the challenges of real-life clinical practice can prove difficult to do. Over the last couple of years, different machine-learning approaches have been proposed to score anti-tumor immunity, resulting in a variety of TIL biomarkers with potential clinical applicability. However, many of these machine-learning-derived TIL biomarkers lack the broad validation essential for clinical adoption. Furthermore, there is a need for studies comparing various AI models focusing on both analytical and prognostic validity.

In this study, we compared the analytical and prognostic validity against invasive disease-free survival of ten AI-based TIL assessment models in TNBC tumors from the prospective Swedish SCAN-B study. Seven models (i.e. KNN, RT, and NN) were constructed within QuPath for automated TILs assessment. Additionally, we employed three pre-trained and validated advanced convolutional neural network (CNN) models, i.e. HoverNet,^29^ CellViT,^30^ and Abousamra et al.,^31^ as independent state-of-the-art predictors. When examining the analytical performance of different AI model methodologies, we found a moderate–good correlation among the different AI models even when these models were trained on an extensive number of samples. Models with similar architecture (NN10-NN50) showed high correlation irrespective of the training samples.

Disparities in the analytical performance are shown when considering the correlation between machine-read TILs scoring and manual assessment of sTILs in internal and external validation. In the internal validation set, all AI models showed a good correlation with the pathologist-read TILs scores, while there was a drop in performance in the external validation set, resulting in only moderate correlation. Furthermore, increasing the number of training samples did not lead to improved correlation results. Therefore, it appears that there are significant differences between internal and external validation results across AI methodologies and training strategies, especially when different slide scanning platforms are used to obtain the images. Our results highlight the importance of assessing model performance across diverse datasets and environments to ensure robustness and generalizability of automated TILs assessment methods.

Predefined and clinically-relevant cut-off of 10%^34^, ^35^, ^36^, ^37^, ^38^ was used to dichotomize TILs scoring of all models. However, observing the TILs score distributions of most models, we found that they significantly differ from the pathologist-read sTILs. We believe that such discrepancies lie in the inherent differences of each method and are due to the following phenomena. First, sTILs calculation is not based on cell-counting but rather is a semi-quantitative measurement of the area that TILs cover based on visual comparison of patient's image and baseline examples, which may lead to over-estimation of the true TILs score per definition. On the other hand, easTILs is a mathematical formula, calculated on the basis of deterministically reproducible cell segmentation data. In contrast, Abousamra's formula calculates the TILs area using whole patches rather than individual cell outlines. This makes Abousamra's method more comparable to the sTILs approach, explaining the similarity in their distributions. Another factor is the cell segmentation algorithm used, which can over-segment or under-segment cells. For instance, watershed-based algorithms in QuPath produce more false-positive cell detections compared to HoverNet and CellViT, often identifying non-cell tissue areas as cells.^29^ Additionally, HoverNet and CellViT achieve finer cell segmentation,^29^ reducing thus the nucleus and cytoplasm areas. This directly affects the easTILs formula, resulting in lower TILs scores. Therefore, when comparing different AI models in prognostic validity, we recommend using a continuous TILs score in the survival analysis instead of cut-off-based categorical scores to enhance comparability.

Despite the variations in the analytical performance, the prognostic potential, using IDFS as clinical endpoint, of digital TILs is demonstrated for the vast majority of the AI models except for CellViT^30^ and Abousamra et al.^31^ All the significant models showed comparable and overlapping hazard ratios in the entire validation set as well as in the chemotherapy-administered subgroup. Interestingly, even models with lower training samples showed robust prognostic potential. We believe that this is due to the fact that host anti-tumor immunity as a biomarker (measured by TILs) is very robust, which can lead to even less extensively trained models performing adequately in assessing prognostic outcomes. However, training on very small datasets, as is the case of the own-developed models in our study, increases the risk of overfitting, limiting the generalizability of these models to broader clinical contexts. Thus, it is important to train such models on sufficiently large and diverse datasets to ensure both their prognostic validity but just as importantly their analytical robustness and generalizability.

A limitation of this study is the fact that we didn't train a regression-based model for directly enumerating TILs scores, which would add to the diversity of the examined methodologies. However, we believe that training regression-based models would be inferior to patch- and cell-classification strategies, as it would directly depend on the pathologists' sTILs as ground truth and thus they would inherit all its limitations.

Clinical adoption also requires AI models to thoroughly explain their decision-making process, particularly in cases of false results. Most AI models in this study use a cell segmentation paradigm to calculate the TILs score. This approach offers a unique advantage over patch classification and regression-based methodologies, with respect to its intuitive interpretability. Clinicians can directly observe detected cells for misclassifications or unidentified cells, making the process more transparent and reliable. Such transparency is crucial for gaining clinician trust and ensuring the model's practical utility in real-world clinical settings.

Another supposed limitation would be the fact that we cannot measure the pathologists' agreement on scoring sTILs, as there is no overlapping subset in this study's cohorts. However, we consider this an advantage of our study, as it mirrors real-life clinical practice and captures any inherent variability, as discussed in the introduction section.

The retrospective analysis of prospectively collected data introduces an additional limitation, in that it prevents us from providing evidence for the clinical utility of AI models scoring TILs. Level 1 evidence for clinical utility of pathologist-read TIL scoring has recently been published^39^ but it remains to be seen if this publication will affect the adoption or reimbursement for AI-based TIL scoring. Note that this is one of the first studies to compare the analytical- and prognostic validity of several independent AI models scoring TILs in an independent external validation setting. Further validation on the large publicly available TCGA dataset was unfortunately not possible, since it had been used to train the independently pre-trained deep learning models used in this study.

Clinical application of such models requires their validation in a well-defined but broad context, that captures the clinical reality of breast cancer tumors. Given that many studies validate their models in a very narrow context, and that many, especially those implemented with a copyrighted service, are not being released publicly, it may be interesting to develop a way to benchmark them in a unified environment that would not compromise their intellectual property rights. Such would include the development of a diverse and inclusive dataset and the infrastructure to validate any model (open source or not) against it, for which an independent framework would perform the calculations to ensure reliability in the results. This would not only increase the trustworthiness of the models but would also aid in their clarity and in identifying the advantages or drawbacks of each.

In conclusion, we demonstrate the existence of variability in both analytical- and prognostic validity against invasive disease-free survival between different AI TIL scoring models tested in an independent prospective cohort. There is a critical need for an accessible, large, multi-centric benchmark dataset encompassing several clinical trial cohorts to prove the prognostic validity and utility of AI TILs scoring models and to ensure comparability between different AI methodologies before clinical implementation. The multi-institutional CATALINA challenge study^40^ is underway and may be suitable for this purpose.

## Contributors

JMV and BA were responsible for the concept of the study. BA and JH were responsible for the study design. JMV, NT and BA had full access to all the data and verified the underlying data. JMV, NT and BA were responsible for the data collection, curation and their quality control. JMV, NT and BA performed the verification and quality control of the algorithms. JMV and NT carried out the technical and statistical analysis. All authors were involved in the interpretation of the data results. JMV, NT and BA drafted the manuscript. All authors were involved in reviewing and editing the manuscript. All authors approved the final manuscript for submission.

## Data sharing statement

The data are not publicly available due to restrictions by Swedish and European law, in order to protect patient privacy. Data are available for researchers with relevant ethical approvals and who meet the criteria for access to confidential data upon request.

## Declaration of interests

JH has obtained speaker's honoraria or advisory board remunerations from Roche, Novartis, Pfizer, EliLilly, MSD, Gilead, Sakura and has received institutional research support from Roche, AstraZeneca, MSD and Novartis. JH is a co-founder and shareholder of Stratipath AB. AB has received honoraria from Gilead for participation in advisory board meetings and has received institutional honoraria for lectures and participation in advisory board meetings from Pfizer, Roche, Novartis and Elli Lilly. AB is a co-founder, shareholder, and CEO of SACRA Therapeutics AB. DLR has served as a Consultant/Advisor to Astra Zeneca, Cell Signaling Technology, Cepheid, Danaher, NextCure, PAIGE.AI, Regeneron, and Sanofi. Cepheid, NavigateBP, NextCure, and Leica currently fund, or have previously funded, research in his lab. RS serves on an Advisory Board and/or has a consultancy role for BMS, Roche, Owkin, Astra Zeneca, Daiichi Sankyo and Case45. RS has received research funding by Roche, Puma, Merck and BMS. RS has received travel and congress-registration support by Roche, Merck, BMS, Daiichii Sankyo and AstraZeneca. All the other authors had no potential conflicts of interest to disclose.

## Appendix A Supplementary data

Supplementary Figure S1 Clinical characteristics of all patients included in each training set of the NN10-50 classifiers, as well as the internal validation subset of the Yale cohort.
