# Supplementary material for: The analytical and clinical validity of AI algorithms to score TILs in TNBC: can we use different machine learning models interchangeably?
Source: eClinicalMedicine. 2024 Nov 15;78:102928. doi: 10.1016/j.eclinm.2024.102928 (PMC11615110; doi:10.1016/j.eclinm.2024.102928)
Supplement: Supplementary Figure S1 — Clinical characteristics of all patients included in each training set of the NN10-50 classifiers, as well as the internal validation subset of the Yale cohort. [file mmc1.pdf]

**Characteristic**	**All**, N = 79[1]	**Train_10**, N = 10[1]	**Train_20**, N = 18[1]	**Train_30**, N = 27[1]	**Train_40**, N = 35[1]	**Train_50**, N = 43[1]	**Validation**, N = 36[1]	**p-value**[2]
Age								0.9
<50	21 (27%)	3 (30%)	4 (22%)	7 (26%)	7 (20%)	9 (21%)	12 (33%)	
>=50	58 (73%)	7 (70%)	14 (78%)	20 (74%)	28 (80%)	34 (79%)	24 (67%)	
Size								>0.9
<=20	37 (47%)	6 (60%)	9 (50%)	13 (48%)	17 (49%)	21 (49%)	16 (44%)	
>20	42 (53%)	4 (40%)	9 (50%)	14 (52%)	18 (51%)	22 (51%)	20 (56%)	
Continuous Size	21 (15, 31)	16 (15, 29)	22 (15, 30)	21 (15, 30)	21 (15, 30)	21 (15, 30)	22 (15, 32)	>0.9
Nodal Status								>0.9
Neg	52 (66%)	7 (70%)	11 (61%)	17 (63%)	22 (63%)	28 (65%)	24 (67%)	
Pos	27 (34%)	3 (30%)	7 (39%)	10 (37%)	13 (37%)	15 (35%)	12 (33%)	
Grade								0.2
2	17 (22%)	3 (30%)	7 (39%)	11 (41%)	11 (31%)	12 (28%)	5 (14%)	
3	61 (78%)	7 (70%)	11 (61%)	16 (59%)	24 (69%)	31 (72%)	30 (86%)	
Unknown	1	0	0	0	0	0	1	
Administered Chemotherapy								0.9
NoChemo	12 (21%)	3 (38%)	4 (27%)	6 (25%)	8 (26%)	9 (24%)	3 (15%)	
Chemo	46 (79%)	5 (63%)	11 (73%)	18 (75%)	23 (74%)	29 (76%)	17 (85%)	
Unknown	21	2	3	3	4	5	16	
OS	10.6 (6.3, 16.4)	10.9 (8.3, 11.6)	9.9 (5.6, 12.2)	10.8 (5.9, 14.6)	11.6 (6.8, 15.7)	11.1 (6.8, 15.7)	9.7 (6.1, 17.0)	0.8
Unknown	2	2	2	2	2	2	0	

[1] n (%); Median (IQR)

[2] Fisher's exact test; Kruskal-Wallis rank sum test
